# Focused on the Family: Development of a Family-Based Intervention Promoting the Transition to Adult Health Care for Adolescents with Type 1 Diabetes

**DOI:** 10.3390/children11111304

**Published:** 2024-10-28

**Authors:** Jaclyn L. Papadakis, Madeleine C. Suhs, Alexander O’Donnell, Michael A. Harris, Lindsay M. Anderson, Kimberly P. Garza, Lindsey Weil, Jill Weissberg-Benchell

**Affiliations:** 1 Ann & Robert H. Lurie Children’s Hospital of Chicago, Chicago, IL 60611, USA; 2 Department of Psychiatry and Behavioral Sciences, Northwestern University Feinberg School of Medicine, Chicago, IL 60611, USA; 3 Harold Schnitzer Diabetes Health Center, Oregon Health & Science University, Portland, OR 97239, USA; 4 Phoenix Children’s Hospital, Phoenix, AZ 85016, USA; 5 Department of Sociology and Public Health Studies, Roanoke College, Salem, VA 24153, USA; 6 Children’s Health Council, Palo Alto, CA 94304, USA

**Keywords:** adolescents, family, intervention, transition to adult healthcare, type 1 diabetes

## Abstract

**Background/Objectives:** There is minimal evidence for current interventions promoting the transition to adult healthcare for youth with type 1 diabetes (T1D). Few interventions exclusively target modifiable individual and family-based factors that contribute to transition readiness. The purpose of this paper is to describe the development of Behavioral Family Systems Therapy for Diabetes Transition (BFST-DT), a virtual family-based transition readiness intervention for adolescents with T1D. **Methods:** The development of BFST-DT occurred in three phases. In phase 1, focus groups with adolescents and young adults with T1D, their caregivers, and pediatric and adult diabetes providers were conducted to assess perspectives on common family challenges surrounding diabetes management and the transition to adult healthcare. In phase 2, focus group data were used to create video vignettes to be used as part of the intervention. In phase 3, BFST-DT was created through the adaptation of a previous evidence-based family intervention for families of adolescents with T1D. **Results:** BFST-DT is a virtual, 6-month family-based intervention involving four multi-family group meetings and six individual family meetings. It targets the modifiable and reciprocal interactions among individual and family transition readiness factors. **Conclusions:** BFST-DT is the first family-focused intervention promoting transition readiness in adolescents with T1D and is currently being tested. Intervention development benefits from prioritization of engagement with patients, caregivers, and providers, as their perspectives are invaluable for creating interventions that are relevant and acceptable to communities.

## 1. Introduction

Diabetes care and resulting glycemic control are poorly managed during late adolescence and emerging adulthood, making it a high-risk time for individuals with type 1 diabetes (T1D) [1,2]. Up to 60% of emerging adults with T1D do not transfer from pediatric to adult diabetes care successfully and instead experience declines in clinic attendance, limited access to necessary medical services, adverse medical outcomes (e.g., failure to meet glycemic targets) [3,4,5], and psychosocial challenges (e.g., depression) [6,7,8]. Emerging adults are the least likely out of all age groups to achieve glycemic targets. As few as 17% do so, with registry-based studies suggesting hemoglobin A1c peaks during the early phase of emerging adulthood (around the ages of 18 and 19) [1,2,9].

The negative outcomes seen during emerging adulthood may result from the convergence of multiple transitions that typically occur during this period [10,11]. Emerging adulthood is a distinct developmental period characterized by increased independence, instability, risk-taking, family discord, and milestones such as entering the workforce or attending college [12,13]. Emerging adults with T1D must manage these normative changes and challenges while also assuming more diabetes-related responsibility from caregivers and transferring from pediatric to adult healthcare [13].

Transition readiness is a multi-component, multi-systemic construct that includes the individual and family skills and conditions believed to prepare emerging adults for successful diabetes management in the context of becoming independent from their caregivers in their diabetes management and in the context of transferring from pediatric to adult healthcare [14]. Research has revealed how individual and family transition readiness factors are related to important medical and psychosocial outcomes during the emerging adulthood period and following the transition to adult healthcare [15,16]. Social–ecological and adolescent development theoretical models of transition readiness recognize the significant inter-relationships among these key domains and highlight why transition readiness assessment and preparation is recommended during the adolescent years, prior to emerging adulthood [12,17,18,19].

There is minimal evidence for current transition readiness interventions in T1D [20]. The transition readiness interventions that do exist are largely clinic-based and focus on clinician–patient encounters or health system factors that are less modifiable [20,21,22,23,24]. To our knowledge, no interventions exclusively target modifiable individual and family-based factors that contribute to transition readiness, nor measure family-based outcomes [25,26,27]. Indeed, at the time of this writing, there is only one published intervention for transition in T1D that involves family members [28]. Since adolescents are particularly sensitive to family influences around individual health behaviors [15], building collaborative transition readiness goals within a supportive family context will likely lead to improved outcomes. In fact, young adults who have strong family support when they are adolescents demonstrate better psychosocial functioning broadly, which is associated with transition readiness [25].

### Current Objective

The purpose of this paper is to describe the development of Behavioral Family Systems Therapy for Diabetes Transition (BFST-DT), a virtual family-based transition readiness intervention for adolescents with T1D that was adapted from an existing empirically supported family-based intervention for youth with T1D, Behavioral Family Systems Therapy for Diabetes (BFST-D) [29]. BFST-DT is the first intervention designed to target the modifiable and reciprocal interactions among individual and family transition readiness factors. It is the first intervention that capitalizes on allowing adolescents the opportunity for guided practice of the skills necessary for transition while still in their caregivers’ care. This paper describes the development of BFST-DT and highlights how patient, caregiver, and provider engagement is critical to intervention development to ensure interventions are applicable and acceptable and can be implemented successfully [30,31,32].

## 2. Methods and Results

### 2.1. BFST-DT Development Overview

BFST-DT was created through the adaptation of Behavioral Family Systems Therapy for Diabetes (BFST-D), an intervention for families of adolescents with T1D experiencing high levels of conflict and poor metabolic control [33]. The current research team, which includes leading experts in diabetes transition and one of the co-developers of BFST-D, aimed to adapt BFST-D by adding transition-specific content and components. It was expected that BFST-DT would lead to the same improvements in metabolic and psychosocial outcomes as BFST-D.

The adaptation was informed by (1) recommendations by the National Diabetes Education Program [34], the American Diabetes Association [35], the GotTransition coalition [36], and the American Academy of Pediatrics [37]; (2) the research literature on transition in T1D; and (3) patient and provider perspectives collected via focus groups with adolescents and young adults with T1D and their caregivers and focus groups with diabetes providers. Engaging patients and providers in intervention development can ensure that the intervention is aligned with the targeted population’s needs [31,32] as well as improve the intervention’s future integration into the clinic setting [30]. What follows is a description of the iterative adaptation process that was centered around patient, caregiver, and provider engagement.

### 2.2. Phase 1: Patient, Caregiver, and Provider Perspectives on Transition Readiness

All research activities described here were reviewed by the Institutional Review Board of a pediatric academic medical center in a large city in the Midwest. To inform the development of transition-specific content, focus groups were conducted with each of the following four groups: adolescents with T1D and a primary caregiver, young adults with T1D who already transferred their diabetes care to an adult provider and a primary caregiver, pediatric diabetes providers, and adult diabetes providers. Each focus group addressed participants’ perspectives regarding common challenges to adolescent and young adult management of T1D, including how families work together on diabetes management and navigate the transition process. Due to time constraints of the funding supporting focus group data collection, convenience sampling was used. Informed consent was obtained for adult participants, and parental informed consent and adolescent informed assent were obtained for adolescent participants. All participants were provided a USD 25 gift card for their participation.

Adolescents with T1D and their caregivers were recruited in-clinic via flyers or by being approached by study staff. In total, 10 adolescents (*M*age = 16.3 years; *n* = 5 (50%) were female; *n* = 5 (50%) were White) and their caregivers participated. Example focus group questions included: *What are some of the things around daily diabetes care that lead to disagreements or frustrations in your family? What concerns do you have about making the transition to adult care?*

Young adults with T1D who had recently transferred their care to an adult provider and their caregivers were identified by their pediatric diabetes provider and recruited via phone call. In total, four young adults (*M*age = 20.3 years; *n* = 2 (50%) were female; *n* = 2 (50%) were White) and their caregivers participated. Example focus group questions included: *In high school, where did you and your caregivers get stuck the most when trying to communicate about diabetes? Tell us about what worked well and what was really challenging about transferring care?*

The pediatric diabetes providers were recruited via email invitation from the pediatric academic medical center. In total, nine pediatric diabetes providers participated (*n* = 9 (100%) were female; *n* = 6 pediatric endocrinologists; *n* = 2 diabetes nurse educators; *n* = 1 nurse practitioner). Example focus group questions included: *What are some of the areas of conflict that you see between caregivers and adolescents around diabetes care? What information or specific skills do you feel that families need to transfer care with minimal difficulties?*

The adult diabetes providers were recruited via email invitation from adult academic medical centers. In total, four adult diabetes providers participated (*n* = 4 (100%) were female; *n* = 4 adult endocrinologists). Example focus group questions included: *What aspects of family functioning relate to challenges or difficulties in transferring to an adult care provider? After a patient transfers to your care, from a pediatric provider, what are the biggest struggles or challenges for individuals regarding their diabetes care?*

All focus groups were audiotaped, transcribed, deidentified, and uploaded to NVivo 12.0 for coding. Three coders first read all the transcripts to familiarize themselves with the data. Following this review, two transcripts were coded using a hybrid thematic analysis approach [38,39]. This approach includes both inductive and deductive coding, which allows coders to capture emerging constructs as they appear in the data [40,41]. An initial codebook with the a priori codes was established, and the team reviewed a small number of transcripts. After the review, the coding team discussed the codes, added additional codes if needed, and corrected coding discrepancies. Coders met weekly to discuss the transcript content and any coding discrepancies. After the coding was complete, the data were sorted into seven themes: concerns about transition, barriers and difficulties during transition, roles and relationships, transition knowledge and skills, problem-solving difficulties and strategies, and communication difficulties and strategies. See Table 1 for example quotes for each theme.

### 2.3. Phase 2: Creation of Intervention Video Content and Focus Group Feedback

Appreciating that individuals and families may learn and engage best in differing ways, and in an effort to increase interest, engagement, and relevancy of the intervention content, the results from the focus groups were used to write scripts for short-length (under 5 min) videos depicting vignettes of common family interactions around diabetes management and transition. Research has shown that using videos enhances youth participants’ engagement in digital mental health interventions [42]. Three research team members independently reviewed focus group results to generate ideas for video content. The goal was to attempt to generate two video scripts for each of the seven themes identified from focus group data (as detailed in Table 1), although some themes provided ample content to support multiple scripts, and others did not. In addition, it was a goal to have video scripts reflect both family challenges surrounding diabetes management and transition, aligned with the aim of the focus groups. Through several meetings and discussions, 12 video scripts were drafted and brought to the entire team for review, discussion, and editing. Six videos focused on the transition to adulthood or the transition to adult diabetes care and included young adults with T1D and their caregivers and providers navigating or reflecting on the transition process. The other six videos focused on common challenges that arise between teenagers and their caregivers around diabetes management and demonstrated adaptive and less adaptive family interactions.

With the finalized scripts, pilot videos were filmed with research team members participating as the actors. Once pilot videos were recorded, a subset of the participants from the initial focus groups were invited to participate in a second-round focus group to watch the pilot videos and offer feedback. Participants were reconsented following procedures described previously. In total, two adolescents and their caregivers, two young adults and their caregivers, five pediatric diabetes providers, and two adult providers participated. Example focus group questions included: *What are your reactions to the video? What would you change? Is this video helpful in addressing transition readiness; why or why not? Is this video helpful in improving family interactions around diabetes; why or why not?*

Focus groups were audiotaped, transcribed, and de-identified. Transcripts were coded and then thematically analyzed by multiple research team members. Three themes emerged: (1) watchability of the videos—identification of entertaining aspects, desire for shorter videos, and suggestions about using different scenes or set-ups; (2) the realistic nature of the video content—removing unrealistic dialogue or characteristics; and (3) the relevancy of the content—suggestions about relatability to content, using language that was more inclusive, and showing a variety of teen and caregiver reactions.

These data informed script revisions and resulted in the deletion of one of the twelve videos due to lack of relevancy. The research team then hired a professional video production company and professional actors to film the new videos. The research team co-directed all filming and participated in the editing of the videos to ensure fidelity to focus group input and content accuracy.

### 2.4. Phase 3: Adaptation of Intervention and Focus Group Feedback

Simultaneous with the filming of professional videos, the research team adapted the BFST-D intervention into BFST-DT. Behavioral Family Systems Therapy (BFST) [43] is a structured therapy for enhancing parent–adolescent communication and problem-solving skills that have been shown to be effective with distressed families of healthy and chronically ill adolescents. BFST consists of three main components: problem-solving, communication skills, and cognitive restructuring. Behavioral Family Systems Therapy for Diabetes (BFST-D) was adapted from BFST for adolescents with T1D experiencing high levels of conflict and poor metabolic control [33]. It was created by incorporating diabetes-specific components (e.g., advanced education on self-monitoring blood glucose and parental simulation of living with diabetes) into the preexisting foci of problem-solving, communication, and cognitive restructuring. It consists of 10 weekly meetings over a 12-week period between a family (adolescents aged 11–18 years and their caregiver(s)) and a licensed psychologist. BFST-D has been proven to improve diabetes outcomes and family functioning via in-person and telehealth modalities [44,45,46]. However, BFST-D was not specifically designed to promote transition readiness and does not focus on the individual or family factors that are key to transition readiness. Therefore, it was adapted into BFST-DT by making changes to the telehealth delivery, targeted age group, time commitment, content, and modalities.

#### 2.4.1. Telehealth Delivery

BFST-D was originally delivered in person but was adapted to be delivered via telehealth in its most recent iteration [45]. BFST-DT was also designed to be delivered virtually, recognizing the growing demand and benefit of virtually delivered healthcare. Indeed, delivering an intervention via telehealth offers numerous advantages over traditional intervention approaches, as it can overcome barriers to care for families from under-resourced backgrounds [47,48] such as geographical proximity to the main clinical site, transportation time and costs, and missed work or school. Telehealth is also an advantageous approach given the likelihood of recurrent public health threats such as the COVID-19 pandemic. Importantly, studies confirm the feasibility and effectiveness of telehealth with youth with diabetes and their families [45,47,49,50,51].

#### 2.4.2. Targeted Age Group

BFST-DT was adapted to target juniors and seniors in high school. These late adolescent years are an ideal time for transition-readiness interventions given that adolescents typically are assuming more responsibility and becoming more independent yet are still receiving the support of their families. Also, focusing on late adolescents was expected to maximize interest and engagement, as it is more likely that adolescents and caregivers alike will already be thinking about the coming transition, as compared to early adolescent years.

#### 2.4.3. Time Commitment

In an effort to reduce potential time commitment burdens, BFST-DT was designed to be delivered over 10 meetings in a 6-month period: a 2 h multi-family group meeting occurs once per month during the first four months, and a 1 h individual family meeting occurs once per month for all six months. This number of sessions has been supported in the literature [52,53].

#### 2.4.4. Content and Modalities

**Figure 1:**
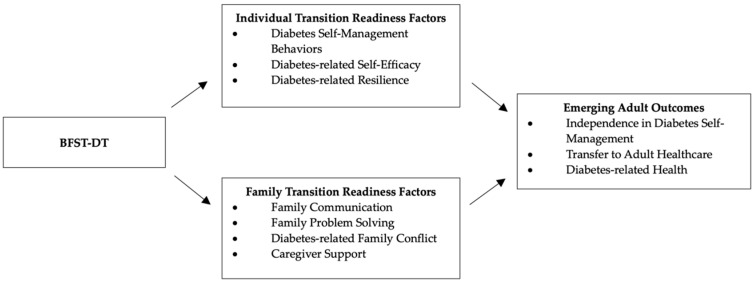
BFST-DT Conceptual Model.

In order to promote transition readiness, BFST-D was adapted to retain the focus on family problem-solving, communication skills, and cognitive restructuring but with additional recognition of the individual and family skills needed to successfully manage the transition process. BFST-DT does this by attending to the reciprocal interactions between diabetes self-management behaviors, diabetes-related self-efficacy, diabetes-related resilience, diabetes-related distress, family communication, family problem-solving, diabetes-related family conflict, and caregiver support. See Figure 1 for the conceptual figure.

BFST-DT incorporates multiple modalities to improve content delivery and engagement. This intervention is designed to be delivered at multiple levels through both multi-family group-based meetings and individual family meetings, with all meetings facilitated by the same psychologist. Research supports the effectiveness of multi-family groups for youth with diabetes [52] and multi-family group meetings are less expensive to conduct than single-family sessions [54], increasing the potential for dissemination into real-world settings. Recognizing that individuals and families may learn and engage best in differing ways, a variety of modalities are utilized in the multi-family group programming including didactic learning, group discussions, discussions within individual families, role-play exercises, and the vignette videos. After providing information about the meeting’s main topic (e.g., family problem-solving), video vignettes are presented with demonstrations of effective and ineffective problem-solving, transition-focused conversations, and communication. Likewise, role-play exercises are conducted to provide a setting for practicing the newly learned skills. See Table 2 for details on the content of multi-family group meetings.

While all families receive the family-based content in the multi-family groups (a universal intervention component), each family also receives individually tailored content through the individual family meetings, which are focused on an individual family’s goals for increasing adolescent transition readiness. At the end of the first multi-family meeting, families are asked to select transition-related goals from the Readiness of Emerging Adults with Diabetes Diagnosed in Youth (READDY) tool [55]. These transition-related goals are the primary focus during individual family meetings. Importantly, the key concepts covered in the multi-family group meetings are reviewed and reinforced during the individual family meetings. Importantly, the individualized content allows the intervention to be shaped to the cultural expectations and values of each family, as it is the family’s goals that are prioritized.

#### 2.4.5. Final Focus Groups

With the newly adapted intervention design and content, including the professional videos that are part of the intervention content, two final focus groups were conducted to ensure the adapted intervention was reflective of patient and caregiver perspectives. Select adolescent and young adult participants and their caregivers from previous focus groups were invited to the focus groups where the lead researcher showed the professional videos and described in detail the overall design and content of the intervention. Participants were re-consented for the final round of focus groups. Participants were asked about their overarching opinions through questions such as the following: *What do you think about this (video/idea)? What would make this (activity) more interesting to you? Would you be willing to participate in this?*

Detailed notes were taken to capture focus group feedback. Specific suggestions were made regarding the professional video content, including dropping some videos for lack of relevancy and interest and re-shooting a video to include content that was more relevant (i.e., adding in content about college partying behaviors into a discussion among young adults). Specific suggestions for revising the intervention included creating handouts to accompany some of the content and providing a transition checklist. Suggestions were also made about specific scenarios or vignettes to present in the multi-family group meetings for group discussion. With this feedback, the research team revised the intervention design and content a final time.

## 3. Summary and Conclusions

The transition to adult healthcare for adolescents with T1D is often marked by worsened glycemic control and limited participation in diabetes care leading to poor illness self-management [1,2]. These youth are not only faced with normative challenges resulting in instability [12,13] but also assume more diabetes-related independence. While there are individual and family factors that are associated with transition readiness and successful transition to adult healthcare [15,16], to our knowledge, there are no interventions that target such factors to better prepare this vulnerable patient population for successful transition of care [25,26,27]. Thus, we adapted an existing evidence-based intervention to create BFST-DT, a virtual family intervention targeting modifiable individual and family factors to improve transition readiness. This is the first family-focused intervention promoting transition readiness in adolescents with T1D. Importantly, we utilized a stepwise approach based on feedback from patients, caregivers, and providers to further develop and refine the intervention through several iterations. Such engagement with patients, caregivers, and providers ensures the relevance and applicability of the intervention to the targeted population [30,31,32]. However, there was a small number of focus group participants overall, and they were recruited via convenience sampling. The development of BFST-DT may have been strengthened by including focus groups of larger numbers, or with patients and providers who reflect more diverse lived experiences, such as those from the broader community who do not receive care through academic medical centers.

BFST-DT is currently being tested via a pilot pre–post intervention design with an iterative mixed-methods assessment of its acceptability, feasibility, and impact on transition readiness factors. Specifically, we are assessing BFST-DT’s effects on individual transition readiness factors of diabetes self-management behaviors, diabetes-related self-efficacy, diabetes-related resilience, and diabetes-related distress. We are also assessing the intervention’s effects on family transition readiness factors including family communication, family problem-solving, family conflict, and caregiver support. To assess feasibility and acceptability, we are conducting focus groups and individual family interviews. We are observing participants during the first two years post-intervention in order to assess the impact of the intervention on longer-term outcomes such as the transition to independent diabetes self-management, the transfer to adult healthcare, and diabetes-related health outcomes. With the results of our current pilot trial, we aim to further refine the intervention based on acceptability, feasibility, and fidelity data before further testing and integration into clinical practice.

Future research would benefit from publishing the details behind intervention adaptation and development. Furthermore, intervention research would benefit from prioritization of engagement with patients, caregivers, and providers, as their perspectives are invaluable to creating interventions that are relevant and acceptable to communities. Adolescents and young adults with T1D and their transition to adult healthcare deserve ongoing clinical and research attention to support this vulnerable but resilient group.

## Figures and Tables

**Table 1:** Themes from initial focus groups with patients and providers.

Theme	Example Quotes
Concerns about transition	“My biggest concern, because he’s getting ready to go off to college is he doesn’t open up and let somebody know that he has diabetes.”—*Caregiver of adolescent* “I think that one of the largest fears we have right now as [son] is getting older and is seeking more responsibility, is seeking more independence, is really how to be able to let go for when he goes to college and have him have a successful college experience while being able to be safe.”—*Caregiver of adolescent*
Barriers and difficulties during transition	“It has just been my job. So yeah, it is very hard when you’re in control to start relinquishing the control and give them [teen] the independence.”—*Caregiver of adolescent* “Because there’s only so much time I have left with him. But I still want to stay on him at the same time, but also give him that space. So just finding that balance, stepping back.”—*Caregiver of adolescent*
Roles and relationships	“I think it’s important that she takes charge of that [ordering supplies], and then I’ll troubleshoot when needed.”—*Caregiver of young adult* “…diabetes is just that–trusting that you raised your child and set an example, but also providing enough support so your child knows you’re still there.”—*Pediatric provider*
Transition knowledge and skills	“I mean we all need help, no matter how old we get in life. And this is just–I don’t know. That’s just what families do! They help and look out for each other.”—*Adolescent* “…we’ve got to do this. He’s got to figure out how to make an appointment every three months. Like a checklist for an 18- or 19-year-old of here’s what you have to do. And handle all on your own.”—*Caregiver of adolescent*
Problem-solving difficulties	“It’s like ‘If your blood sugars aren’t in range all day then you can’t go out with your friends tonight.’ But I am still a teenager, I still want to have fun, and my blood sugar–it isn’t something that like can affect whether I go out with friends or not. It’s just what I am doing.”—*Adolescent*
Problem-solving strategies	“We’ve kind of established like a daily check-in time, which we don’t always do. But… just see how things went that day.”—*Caregiver of adolescent* “So, I think at times when there is an issue, I think what we’re learning to do is just deal with that issue at that time and then give each other some space, and then if we come back to each other when we’re not as filled with anxiety or frustration or anything like that…”—*Caregiver of adolescent*
Communication difficulties	“I just don’t like feeling punished for something that I can’t control at all. I know I can control short-term my blood sugar and stuff, but like I think something that my parents don’t always understand is that unless you have [it]… you have to like be so intricate with it and do all these steps and it’s just unfair.”—*Adolescent*
Communication strategies	“Something that my doctor said to me–my adult doctor–is to just look at it as numbers like mathematically. Like there’s no opinion on it, there’s no tone of voice when you talk about it.”—*Young adult*

**Table 2:** BFST-DT Multi-Family Group Meeting Content.

Meeting and Theme	Content
Multi-Family Meeting #1 Teen and Family Development	**Teens and family development**—e.g., developmental normative changes during adolescence; changing family roles **Teens and diabetes**—e.g., how diabetes management is complicated during adolescence **Family teamwork during the teen years**—e.g., importance of working together; miscarried helping **Video #1: Transition-focused**: parent and college-aged young adult reflect on high school; themes include that conflict is normal, expected, and helpful **Video #2: Transition-focused**: college students meet at a diabetes college event; themes focus on disclosing about diabetes
Multi-Family Meeting #2 Family Problem-Solving	**Family problem-solving**—e.g., autocratic versus democratic problem-solving; shifting in responsibility from parents to teenagers **Video #3: Challenge-focused**: parent and teen communicating about diabetes while they are at a friend’s home; themes include problem-solving so all family members are satisfied with solutions **Video #4: Challenge-focused**: parent wanting to talk with their teen about diabetes, but teen does not; themes include finding the right time to engage in conversations and problem-solving related to diabetes **Video #5: Challenge-focused**: parent and teen reviewing blood sugar numbers; themes include importance of shared identification of barriers to management before jumping to solutions **Video #6: Challenge-focused**: parent and teen at a pediatric diabetes appointment; themes include allowing teen opportunities to build independence **Video #7: Transition-focused**: parent and teen are discussing plans for college; themes include importance of balancing priorities
Multi-Family Meeting #3 Family Communication	**Family communication**—e.g., basic communication skills, nagging, communicating about feelings, difficult conversations **Video #8: Transition-focused**: parents and teens discuss drinking alcohol during college; themes include the importance of having conversations even if difficult **Video #9: Transition-focused**: parent and teen reflect on the transition to college; themes include how communication can differ among multiple family members and can be influenced by family dynamics
Multi-Family Meeting #4 Cognitive Restructuring	**Cognitive restructuring basics**—e.g., the ABC Model; thinking traps

## Data Availability

The data presented in this study are available on request from the corresponding author due to participant privacy.
